# Stability of Whole Brain and Regional Network Topology within and between Resting and Cognitive States

**DOI:** 10.1371/journal.pone.0070275

**Published:** 2013-08-05

**Authors:** Justyna K. Rzucidlo, Paige L. Roseman, Paul J. Laurienti, Dale Dagenbach

**Affiliations:** 1 Department of Psychology, Wake Forest University, Winston-Salem, North Carolina, United States of America; 2 Department of Radiology, Wake Forest University School of Medicine, Winston-Salem, North Carolina, United States of America; University of Maryland, College Park, United States of America

## Abstract

**Background:**

Graph-theory based analyses of resting state functional Magnetic Resonance Imaging (fMRI) data have been used to map the network organization of the brain. While numerous analyses of resting state brain organization exist, many questions remain unexplored. The present study examines the stability of findings based on this approach over repeated resting state and working memory state sessions within the same individuals. This allows assessment of stability of network topology within the same state for both rest and working memory, and between rest and working memory as well.

**Methodology/Principal Findings:**

fMRI scans were performed on five participants while at rest and while performing the 2-back working memory task five times each, with task state alternating while they were in the scanner. Voxel-based whole brain network analyses were performed on the resulting data along with analyses of functional connectivity in regions associated with resting state and working memory. Network topology was fairly stable across repeated sessions of the same task, but varied significantly between rest and working memory. In the whole brain analysis, local efficiency, *Eloc,* differed significantly between rest and working memory. Analyses of network statistics for the precuneus and dorsolateral prefrontal cortex revealed significant differences in degree as a function of task state for both regions and in local efficiency for the precuneus. Conversely, no significant differences were observed across repeated sessions of the same state.

**Conclusions/Significance:**

These findings suggest that network topology is fairly stable within individuals across time for the same state, but also fluid between states. Whole brain voxel-based network analyses may prove to be a valuable tool for exploring how functional connectivity changes in response to task demands.

## Introduction

While much effort has been expended in order to understand localization of function in the human brain over the past thirty years, there is growing interest in using new methodologies to address other important issues in neuroscience. Among these is characterizing the complex interactions that occur within the brain. What parts of the brain are connected to each other structurally and/or functionally? A variety of different approaches have been employed to pursue this question including seed based correlations [1] and independent component analysis [2]. More recently, the application of graph theory to brain imaging data has allowed characterization of complex functional interactions within the entire brain [3]. This paper examines functional connectivity within the human brain using a voxel-based examination of functional Magnetic Resonance Imaging data that allows information from the entire brain to be used in network construction. The stability of the resulting network metrics are assessed within participants across multiple resting states and multiple sessions of an n-back task, a commonly used measure of working memory. In addition, the network metrics are compared across task to determine whether the network topology is significantly altered by cognitive demand.

### Network Science and fMRI

The use of graph theory to help understand complex data sets is known as network science. Network science has been used to characterize complex social networks, the internet, power grids, and fMRI data among other things. In part, network science has worked toward the development of different network metrics in order to measure various aspects of network topology. They are based on the idea that a network is composed of a collection of nodes that are connected by edges, and that these metrics can provide important information about the whole network, individual nodes, and anywhere in between. In neuroscience, these measures have been applied extensively to resting state fMRI data, and have been useful in characterizing normal brain organization and how that changes in disease states such as schizophrenia [4]. One key finding has been that, like many other networks, resting state networks in the human brain exhibit small world properties. Small worldness can be defined in terms of the network having a short average path length and a high average clustering coefficient [5].

Clustering coefficient and path length are commonly used network metrics that measure small worldness, along with local and global efficiency. Degree, another common metric, is a core measure of centrality. Clustering coefficient, *C*, measures the fraction of a node’s neighbors that are also connected to each other and thus provides information about the connectedness of a node’s neighbors. It is related to local efficiency, *E_loc_*, which describes how efficiently information is transferred locally within a sub-network [6]. Path length, *L,* is the smallest number of connections it takes to reach the destination node, and it is related to global efficiency, *E_glob_,* which is the inverse of path length [5]. Global efficiency describes how well information is transferred globally. Degree, *K,* is simply a measurement of the total number of connections for a node. These measures can be averaged for the entire network, and they can also be mapped back onto the brain to examine the location, for example, of the nodes with the highest degree or the highest global efficiency.

### Stability of Whole-brain Network Measures within Task

Given the relative newness of the network science approach to analyzing functional neuroimaging data, it is important to determine how stable such measures are. Do they show stability across time for the same state? Do they change over time when cognitive or emotional state changes? Telesford et al. [7] examined the stability of whole-brain network metrics obtained during two sessions of participants performing a selective attention task. They found evidence of good reproducibility for most, but not all, network metrics. Specifically, intra-class correlation coefficients were high for clustering, path length, global efficiency, efficiency and local efficiency, but not for degree. In addition to looking at the reproducibility of the whole network values, they also looked at reproducibility on a voxel-wise basis. Path length and global efficiency were reproducible across the whole brain, while local efficiency, clustering coefficient, and degree were reproducible primarily in the high degree nodes of the network.

The stability of the default-mode network has been studied as well. The default-mode network is composed of regions of the brain that are active across all normal human brains during a resting or passive state. Research has shown this network to most commonly include the bilateral posterior cingulate/precuneus, inferior parietal cortex, and ventromedial prefrontal cortex, although it can vary to an extent across individuals. This network was first identified by a consistent decrease in activation during goal-directed activities compared to baseline [8], and further research revealed that these brain areas are correlated at rest in adults. Many studies have been able to reproduce the default mode network through co-activation patterns revealed by fMRI time-series of resting state images; however, not many have examined the reproducibility of this network within several resting state imaging sessions over a period of time. One of the few studies that has investigated this idea used independent component analysis to identify the default mode network, and found that it was reproducible both between and within three resting state sessions, particularly in the anterior and posterior cingulate cortex [9]. Additionally, Wang, et al. [10] investigated how the small-world topology of the brain changes after it is influenced by a task. Clustering, path length, global efficiency, and local efficiency all remained stable across the pre- and post- task resting states, meaning that brain intrinsic organization remained consistent during the resting states regardless of preceding task influences. Pyka et al. [11] examined activation with the default mode network before and after an easier classification task and a harder working memory task and found greater increases in activation subsequent to the more demanding task.

Most network science analyses of fMRI data have been applied to resting state data. Thus, most of the analyses have characterized the default mode network [8]. Less is known about how dynamic networks constructed using this approach are when state changes**,** particularly for networks constructed using a voxel based approach. Schroter et al. [12] used a strong manipulation of state by comparing network characteristics between wakefulness and propofol-induced loss of consciousness. Among their findings were the preservation of small-world topology across both states, and a decrease in the clustering coefficient and the proportion of high degree nodes during propofol induced loss of consciousness. In studies where state changes are induced by task, the conclusions have been variable. Using a voxel based network, Eguiluz et al. [13] concluded the topological distribution of brain networks was radically different across tasks. In contrast, Buckner et al. [14] compared visual fixation to a word classification task. The hubs, those nodes with the highest degree, exhibited stability across tasks even though network topology changed, including an increase in the degree (K) in the classification task for nodes in prefrontal and temporal cortex, regions that have previously been linked to performance of the classification task. Although the results of these studies are not directly contradictory since there were considerable methodological differences, it is clear that more research on how various task-related changes in state affect network topology is needed.

### Stability of Regional Network Metrics

Moussa et al. [15] compared network topology for resting state, visual processing, and multisensory processing. They found no significant task-related effects on whole brain network measures. However, they argued that such measures should be used with caution as they merely provide the average measurement of a network and thus may cause significant regional changes to be overlooked. Additional regional statistics may be very helpful in discovering information such as which nodes are influential in a network and which nodes belong to specific communities. When they looked at networks within the auditory and visual cortex, they found network structure in those regions did change significantly across conditions (rest, visual, and multisensory conditions) with respect to clustering, global efficiency, and modularity.

### Network Topology within and between Resting State and a Working Memory Task

The present study is similar in design to that of Moussa et al [15], but changes the task manipulation to a contrast between resting state and a commonly used working memory task, the n-back task. Working memory is an expanded model of short term memory that is involved in higher-level processing such as reasoning and problem solving [16]. It is responsible for the storage and manipulation of information; hence it is frequently relied upon during demanding cognitive tasks. In the n-back task, a stream of stimuli, typically letters, is presented one at a time, and the participant’s task is to indicate whether the current letter is the same as or different from the nth back letter. The memory demands increase as n increases. In the present study, n was set at 2. In addition to contrasting network topology between resting state and working memory, the present study also collected repeated observations of both states so that the stability of network topology could be assessed within task as well as between tasks.

For this study, mean whole brain measures were used to identify how the brain changed as a unit. Subsequent regions of interest (ROI) analyses were then used to further investigate within and between task changes in the connectedness of specific regions of the brain related to the tasks performed. Specifically, the left and right precuneus, as well as the left and right dorsolateral prefrontal cortex (DLPFC), were examined. These regions were chosen from prior literature that identified the dorsolateral prefrontal cortex as a key area in the working memory network and the precuneus region as essential to the default mode network [17], [18]. Research has demonstrated that the precuneus is involved in highly integrated tasks, such as self-processing and visual-spatial imagery tasks [19], and so it is central to the resting state network. The DLPFC, on the other hand, has shown to be essential to a wide range of brain functioning, such as manipulating information, and sustaining attention [20], which are critical to working memory functioning. It was expected that both the default mode and working memory networks would remain consistent across sessions, but that these networks would differ significantly in connectedness and/or efficiency. Networks were constructed for five participants during five resting state and five working memory 2-back sessions that were alternated.

## Results

### Performance on the N-back Task

Participants’ accuracy on the 2-back task ranged from a mean of 86 percent correct to a mean of 95.4 percent correct averaged across the 5 sessions. Overall mean accuracy for the group was 91.3 percent correct. These data indicate that participants were engaged in performing the working memory task.

### Whole-brain Mean Network Metrics

For each participant, whole-brain network metrics were generated for mean *K, E_glob_,* and *E_loc_* for each task and rest session. The mean and standard deviation of each of these metrics were calculated for each subject for both conditions and then averaged as group means (Table 1). 2 (condition) × 5 (session) repeated measures ANOVAs (one for each of these metrics) were used to determine whether any of these metrics varied significantly across the five rest and five task sessions, as well as whether they varied between the two conditions.

**Table 1:** Mean whole-brain metrics for both resting and task states.

Metric	Rest	2-back
*E_glob_*	0.20±0.02	0.20±0.03
*E*_loc_	0.57±0.01	0.54±0.02
*K*	54.19±0.14	54.20±0.12

Global mean metrics across all states were calculated for each subject and averaged as group means. E_glob_, global efficiency; E_loc_, local efficiency; K, average degree; (mean ± SD).

Across sessions, none of the metrics exhibited statistically significant differences (*F = *.53, *p*>.70, *Partial Eta Squared = *.12 for Eglob, *F = *.49, *p*>.70, *Partial Eta Squared = *.11 for Eloc, *and F = *.37, *p*>.82, *Partial Eta Squared = *.08). This result supports the hypothesis that the connectivity of the brain from one resting state to another resting state would remain rather consistent. The same was predicted for the 2-back task state.

While there was no effect of session and no interaction of session with task, a significant main effect of task was observed for local efficiency (Eloc), *F*(1, 4) = 26.6, *p* = .007, *Partial Eta Squared = *.87. The means for *E_loc_* shown in Table 1 indicate that local information transfer was greater for the resting condition than the task condition. The other metrics, *K,* and *E_glob_* exhibited no statistically significant differences between conditions. However, the means of these metrics in Table 1 suggest that all of these metrics, with the slight exception of K and *E_glob_*, were higher in the resting condition.

**Figure 1:**
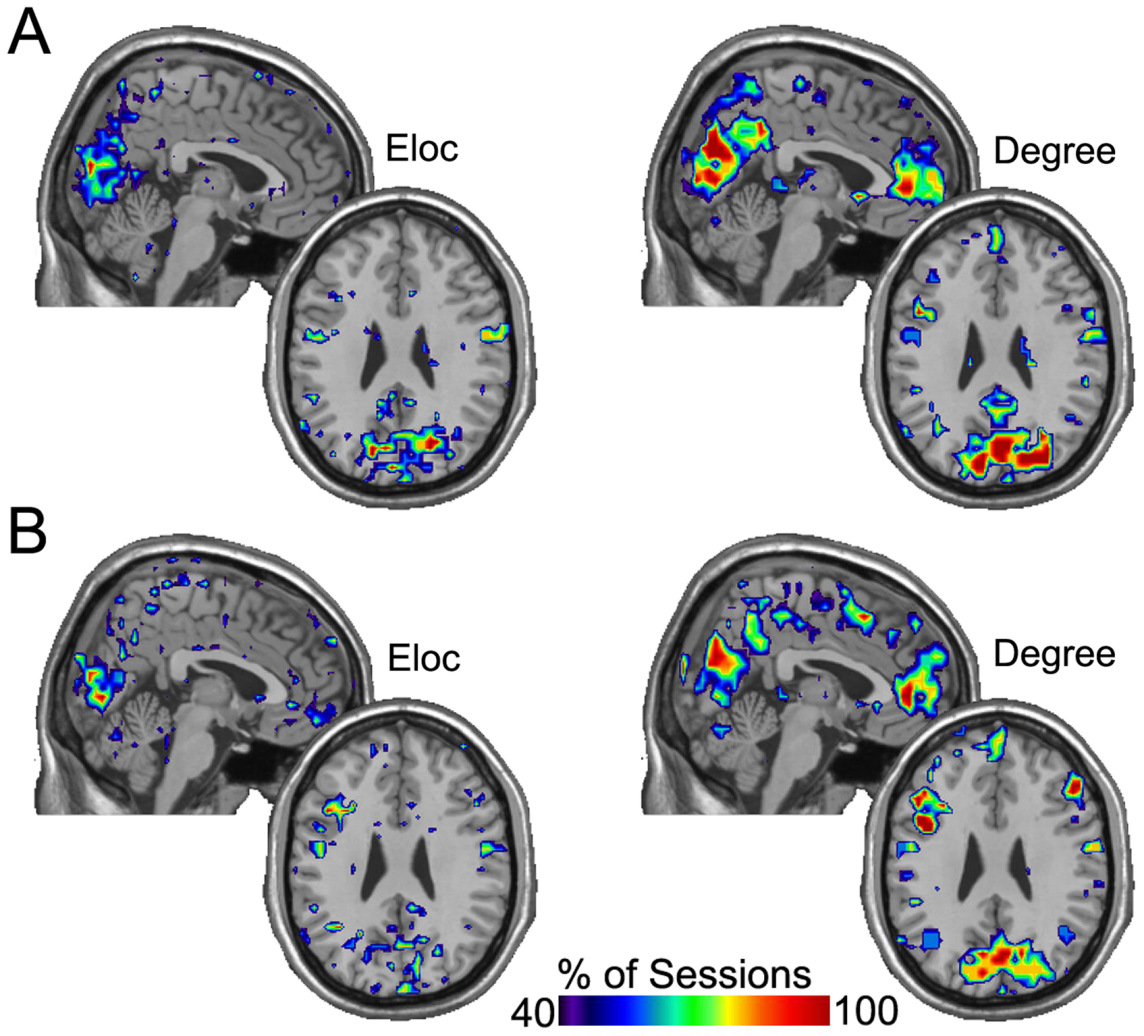
Top overlap maps of local efficiency (*Eloc) and K (Degree)* for a sample participant showing the consistency of the location of nodes in the top 20% across 5 sessions for Rest (A) and n-back (B). The color scale indicates the % of sessions that each area was in the top 20% of nodes. Note that the K maps show greater consolidation and are more task-specific compared to the Eloc maps.

We also examined the spatial distribution of the nodes with the highest local efficiency and degree. For each participant, we first generated maps using the data from the five sessions of resting state and the five sessions of working memory. The maps represent the consistency of the location of the upper 15 percent of nodes for the given network metric. The resulting consistency maps for *Eloc* and K are shown in Figure 1 for a sample participant. The figure shows the areas that were among the top 20% of nodes in Eloc and K in at least 40% of the sessions. First note that K maps exhibit more consolidated regions of consistency compared to Eloc. However, both map types exhibit changes when shifting from resting state to n-back performance. During the n-back task, there was greater consistency of high K and Eloc nodes in the DLPFC compared to rest. At rest, the precuneus exhibited a relatively higher amount of consistency only for K. Eloc was actually not consistently higher in the precuneus in either condition.

**Figure 2:**
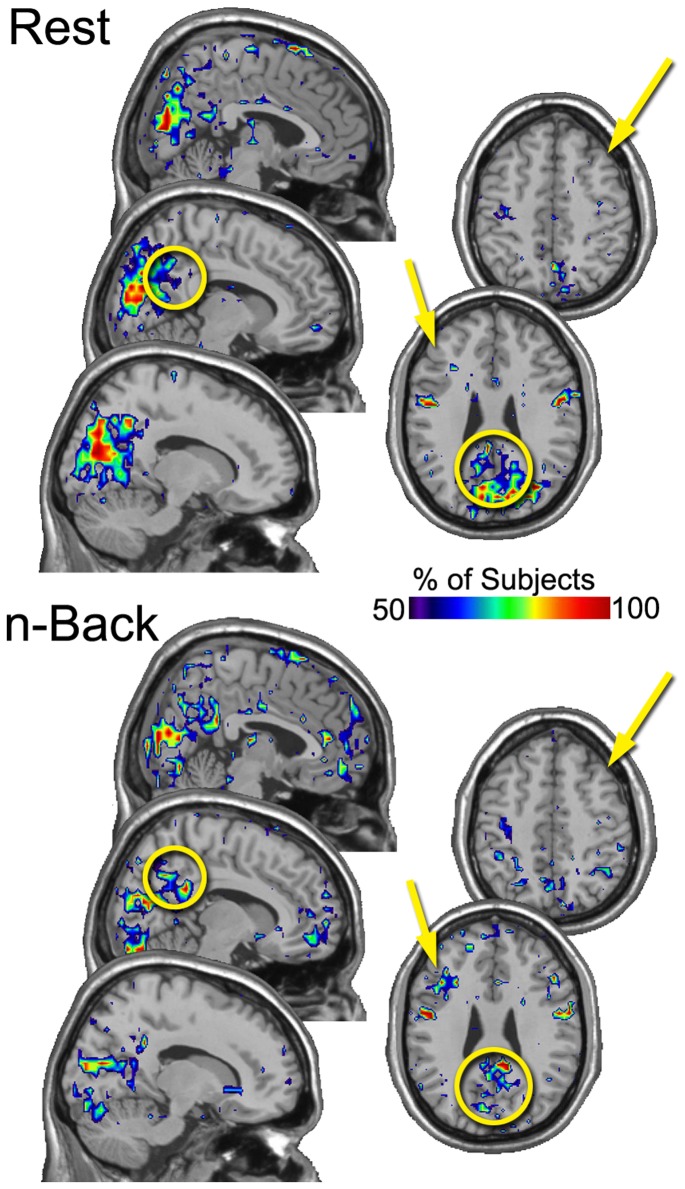
“Meta” consistency maps of *Eloc* showing the percentage of subjects for whom each brain region was consistently the location of high Eloc nodes. The DLPFC exhibited greater consistency across subjexts for the n-back condition compared to the resting condition. The precuneus appeared to have similar consistency between the resting and n-back conditions. The figure includes two axial slices through DLPFC and 3 sagital slices through the precuneus. Only right hemisphere sagital slices are shown as the left side essentially mirrored the right. The arrows point to DLPFC and the circles highlight the precuneus.

**Figure 3:**
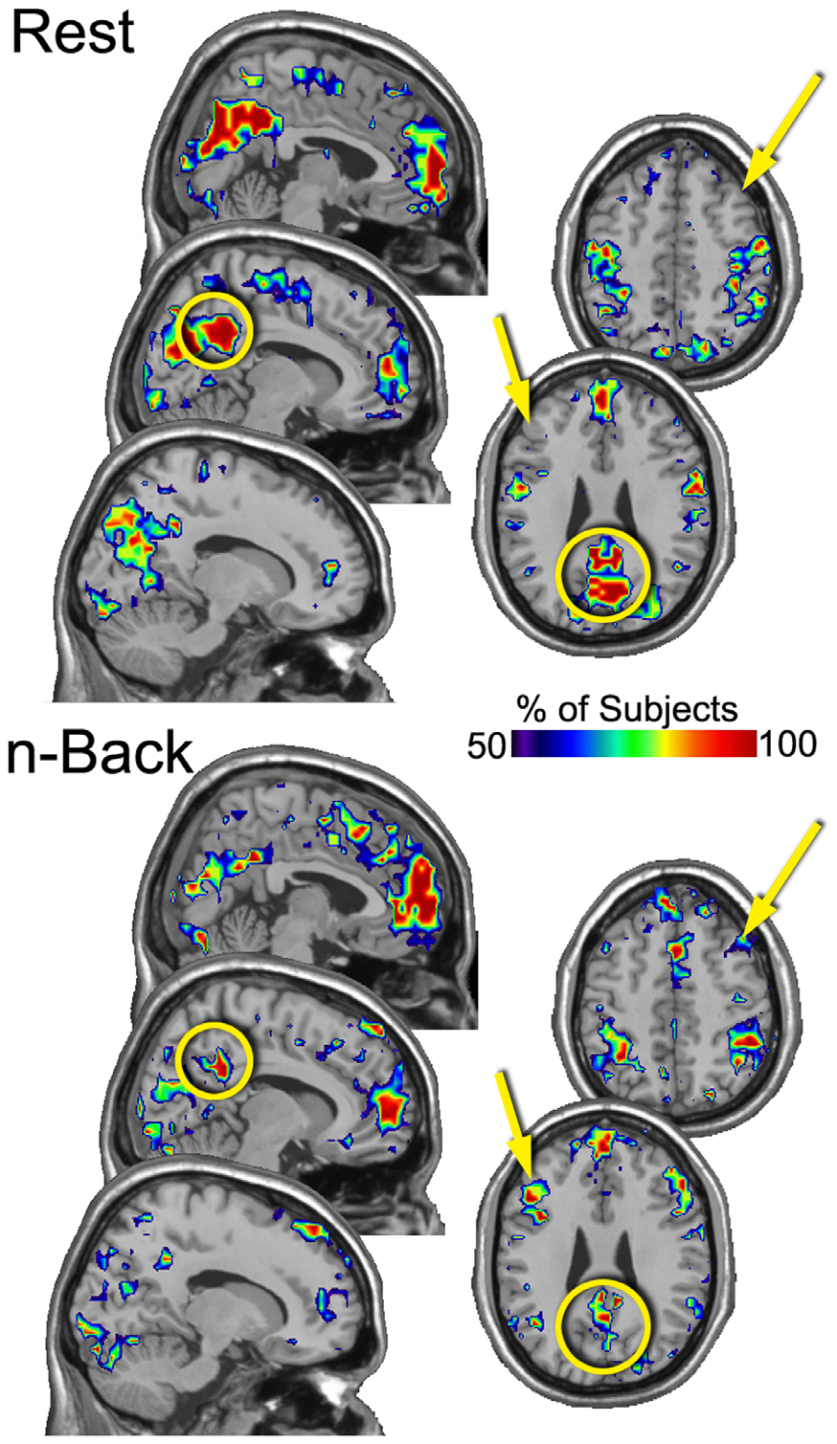
“Meta” consistency maps of K showing the percentage of subjects for whom each brain region was consistently the location of high K nodes. These maps exhibit clear task-specific patterns in the location of the consistently high K nodes. At rest, the precuneus was a major connecting hub in virtually every subject and every session. This consistency drops considerably in the n-back condition. However, the DLPFC becomes highly consistent in the n-back condition. The figure includes two axial slices through DLPFC and 3 sagital slices through the precuneus. Only right hemisphere sagital slices are shown as the left side essentially mirrored the right. The arrows point to DLPFC and the circles highlight the precuneus.

A similar procedure was applied again to the resulting images, this time across subjects, creating in essence a meta consistency map. The maps were thresholded within subjects to include brain regions that were present in at least 3 of the 5 sessions for each condition. The resulting maps for the group show the percentage of people that had a particular brain region in the top 20% in *Eloc* (Figure 2) and K (Figure 3) across 3 or more sessions. These figures clearly demonstrate task-related patterns. As with the individual subject, the Eloc maps show less consistency than the K maps. Nevertheless, it is evident in the Eloc maps that the DLPFC consistently had high Eloc nodes across subjects. The precuneus did not exhibit visually apparent differences a in the consistency of Eloc between rest and n-back performance. However, region-of-interest analyses, presented below, did detect statistically significant condition differences. The K maps show quite dramatic condition differences. The precuneus was highly consistent across participants in the resting condition, indicating that this region was highly connected across resting sessions in nearly all subjects. The DLPFC was not highly connected in the resting condition as indicated by the low consistency levels across subjects. In the n-back condition, the reverse pattern was observed. Specifically, compared to the resting condition, there was higher consistency in the DLPFC and lower consistency in the precuneus.

### Regional Network Metrics

Repeated measures 2×5 ANOVAs were then used to further analyze the statistical significance of the effect of condition on the metrics *K, E_glob_,* and *E_loc_*, but this time within only the DLPFC and the precuneus ROIs. The means and standard deviations of each of these metrics within the precuneus and DLPFC for both the resting and n-back conditions can be seen in Tables 2 and 3, respectively. Once again, there were no main effects of session or interactions between task and session, but there were main effects of task.

**Table 2:** Mean metric values within the precuneus region for both resting and task states.

Metric	Rest	2-back
*E_glob_*	.19±.01	.19±.01
*E*_loc_	.57±.01	0.52±.01
*K*	99.02±39.13	73.65±36.66

Global mean metrics across all states were calculated for each subject and averaged as group means. E_glob_, global efficiency; E_loc_, local efficiency; K, average degree;(mean ± SD).

**Table 3:** Mean metric values within the DLPC region for both resting and task states.

Metric	Rest	2-back
*E_glob_*	0.13±0.01	0.14±0.01
*E_loc_*	0.37±0.01	0.37±0.01
*K*	55.95±35.47	99.72±48.63

Global mean metrics across all states were calculated for each subject and averaged as group means. E_glob_, global efficiency; E_loc_, local efficiency; K, average degree;(mean ± SD).

### Precuneus Results

It was found that average degree differed significantly between the rest and n-back conditions in the precuneus ROI, *F* (1, 4) = 19.12, *p<.02, Partial Eta Squared = *.83. Specifically, the average degree decreased significantly from the resting condition to the working memory condition in the precuneus. Additionally, the results showed that local efficiency differed significantly between the resting state and the working memory condition in the precuneus, *F* (1, 4) = 99.62, *p*<.001, *Partial Eta Squared = *.96. Specifically, local efficiency decreased significantly from the resting condition to the task condition in the precuneus. Global efficiency did not differ significantly between conditions within the precuneus, all *F’s* (1, 4) <1.

**Figure 4:**
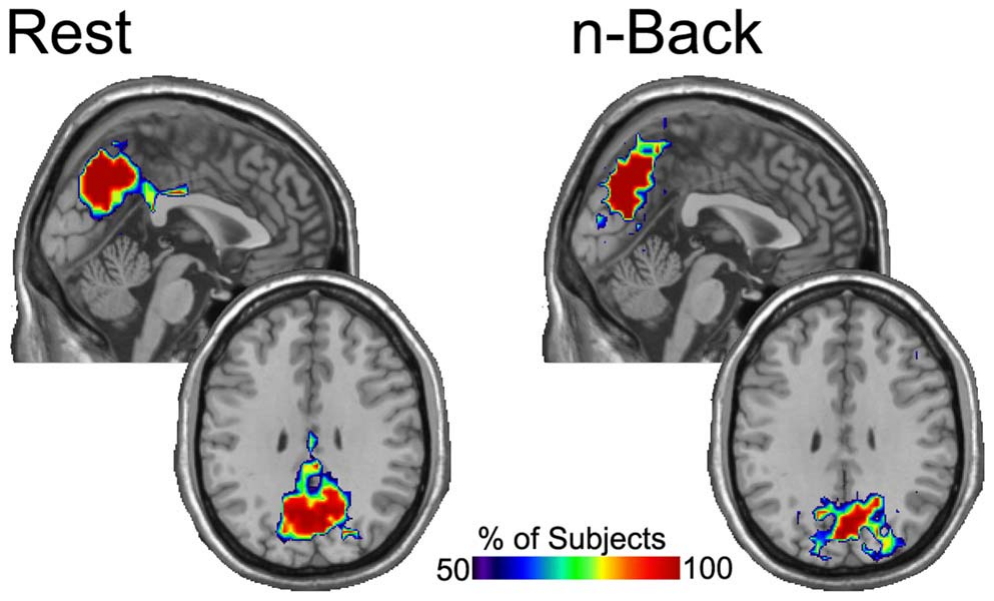
Nodes consistently connected to the top 15 percent high local efficiency nodes in the precuneus across sessions and subjects. The figure demonstrates that these top local efficiency nodes were primarily connected to adjacent brain areas. At rest the focus of connectivity is ventral and includes the posterior cingulate. During n-back the focus of connectivity is more dorsal and includes posterior parietal cortex. A sagital and axial slice through the precuneus are shown.

We explored the meaning of these differences by mapping the network nodes throughout the brain that were connected to the top fifteen percent of the local efficiency and degree nodes within the ROIs. Maps of the spatial overlap of the nodes connected to the high Eloc or K nodes were constructed for each participant across sessions for rest and n-back. The consistency of the spatial location of connector nodes was then determined across participants and threshold of 50% meaning that the majority of participants would have to show that connection for the area to be included. In Figure 4, overlap maps of the nodes connected to the highest local efficiency nodes are shown. The difference between the resting and n-back conditions was quite subtle. The top 15% of Eloc nodes in the precuneus were locally connected to adjacent brain tissue in both conditions. There was a qualitative difference in the location of the main regions of connectivity. In the resting condition the connectivity was focused in the more ventral aspect of the precuneus extending into the posterior cingulate. In the n-back condition the focus of connectivity was in the dorsal precuneus extending back into posterior parietal cortex.

### Dorsolateral Prefrontal Cortex Results

**Figure 5:**
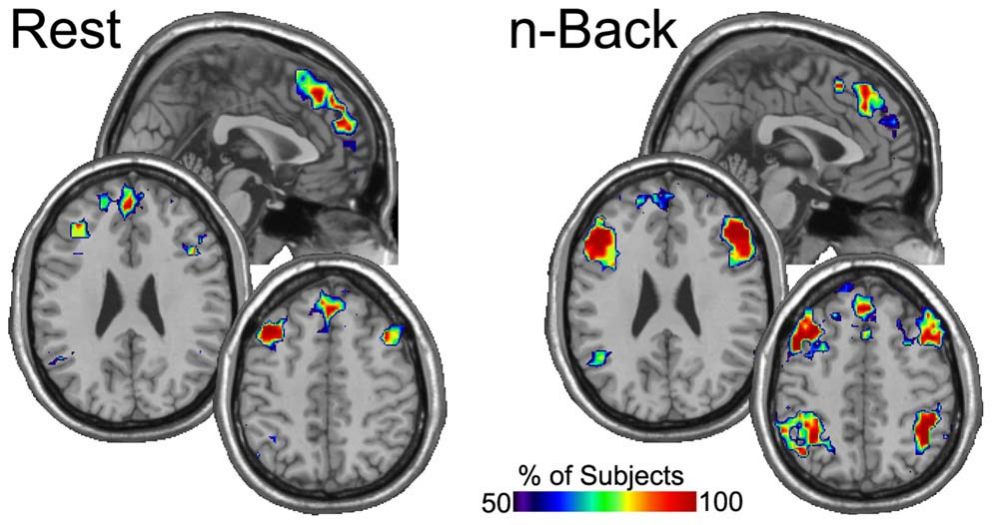
Nodes consistently connected to the top 15 percent high degree nodes in the DLPFC across sessions and subjects. The figure demonstrates that these top degree nodes changed connectivity considerably across conditions. At rest the connections were restricted to frontal cortex. During the n-back conditions connections were consistently present in the lateral parietal cortex. Two axial slices are shown to include the DLPFC and the lateral parietal cortex. The sagital slice is shown to include the precuneus (lacking any consistent connectivity) for comparison to other figures.

It was found that average degree differed significantly between the rest and n-back conditions in the DLPFC ROI, *F* (1, 4) = 57.99, *p<*.01, *Partial Eta Squared = *.935. Specifically, the average degree increased significantly from the resting condition to the task condition in the DLPFC. Conversely, neither global nor local efficiently differed significantly as a function of session, task, or their interaction. The top 15% of the degree distribution nodes in the dorsolateral prefrontal cortex were then determined along with the nodes to which they were connected. Spatial consistency maps for the nodes being connected to were then generated across sessions for rest and working memory for each participant. The top overlap map of these top overlap maps was then determined using a 50% threshold as before. The resulting images are shown in Figure 5 for rest and working memory states. These figures suggest that the top degree nodes in the dorsolateral prefrontal cortex are more connected to posterior occipital areas during working memory and are more in connected to other nodes in frontal cortex while at rest.

The images showing connectivity to the highest degree nodes in the DLPFC exhibit impressive condition-specific patterns. In the resting condition the DLPFC was primarily connected within itself and to the medial aspect of the superior frontal gyrus. Very little connectivity was consistently seen in other brain regions. For the n-back condition the DLPFC was connected to extensively with itself and with the medial superior frontal gyrus. Unlike in the resting condition, there was consistent connectivity between the DLPFC and the lateral parietal cortex, bilaterally. This pattern of connectivity closely resembles the location of the high degree nodes observed in Figure 3.

## Discussion

As mentioned previously, studies such as Buckner et al. [14] concluded that their data suggest that the network structure of the brain remains consistent regardless of the task performed. However, by exploring overall functional connectivity, this study provides further support for the idea that network structure in the brain is actually fluid. The results of this experiment showed that whole brain measures of local efficiency changed significantly from resting state to the working memory task state. The regional measures further demonstrate that there is in fact something fundamental occurring within the regions of the cortical network. Whole brain metrics showed that, on average, degree and global efficiency during the resting state were not significantly different from those of the n-back task state, but the averages of these metrics concealed the significant changes that were occurring in the important cortical regions of the resting and task states- the precuneus and dorsolateral prefrontal cortex, respectively.

Analyses of degree, local efficiency, and global efficiency on these two ROIs showed a statistically significant difference of degree in both the precuneus and DLPFC regions, a finding that had been insignificant across the whole brain. Specifically, connectivity in the precuneus decreased from rest to task, whereas connectivity in the DLPFC increased from rest to task. These results makes sense; other research has shown the DLPFC to be very involved during a working memory task and the precuneus has been repeatedly proven to be crucial to the default mode network [21,19 (respectively)]. Therefore, one would expect the precuneus to increase the number of connections it has with other nodes during a state in which it is highly active (rest), and in turn, it would discard these connections during a state in which it is not as important, such as performing a 2-back working memory task. The opposite would be expected to occur in the DLPFC. This region is responsible for short term storage and manipulation of information, thus it would be expected that this area would extend its number of connections with other nodes while performing a task that required such cognitive activity. Moreover, previous studies have found that degree increased significantly from rest to task in the task-responsible region of interest [15], .

Additionally, local efficiency increased significantly from task to rest in the precuneus, meaning that transfer of information locally within the precuneus was significantly more efficient when the participants were at rest. An increased local efficiency indicates an increase in clustering, thus it is a metric that is capable of identifying regions that are highly specific. In regards to this study, this means that the precuneus region is highly interconnected during the resting state and forms somewhat of a sub-network, but this network loses its efficiency to transfer information to its neighbors when the brain is no longer in a resting state. Although the findings for local efficiency in the DLPFC were not significant, there was a trend for local efficiency to increase from rest to task in this region, suggesting that the precuneus becomes more interconnected during a working memory task.

Lastly, global efficiency did not differ significantly for either of the ROIs; however, the direction of these results was interesting. Global efficiency decreased from rest to task in the precuneus and increased from rest to task in the DLPFC. These results suggest an idea similar to that of local efficiency- a network becomes more efficient in transferring information when it is relied upon to perform a certain action. Increased global efficiency indicates shorter path length, thus these areas of the brain are able to send information across the brain using a fewer amount of nodes when they are required to perform a task. Overall, this study suggests that the connectivity and efficiency of these regions of interest are an important aspect of brain functionality during cognitive states.

Conversely, there were no significant differences in any of the network metrics, global or ROI – based, across resting sessions or working memory sessions. Thus, while the networks are fluid between cognitive states, they also appear to exhibit some stability over time and session for the same state. Taken together, these findings suggest that examining whole brain voxel based networks may provide additional insight into the dynamics of the brain across different states. The conclusions regarding the stability of networks over time and session for the same state are limited by the small sample size of the current study since a larger sample might have produced significant differences between different iterations of the same task due to greater power, although the effects did not even approach statistical significance. More importantly, the finding of differences between states using this approach suggests that further explorations of this issue are warranted.

## Materials and Methods

### Ethics Statement

This research protocol was reviewed and approved by the Wake Forest University School of Medicine Institutional Review Board. Informed written consent was obtained from each participant following the protocol approved by the IRB.

### Participants

Data were collected from five young (23.6±1.67 years old) healthy adults who volunteered to partake in this study. The participants were provided with a MRI Subject Information and Safety Screening Form from the Wake Forest University of Medicine to complete prior to the experiment. The participants were screened again with more detail on the day of the fMRI scan. Any participants who had implants, devices, or objects that would interfere with the fMRI procedure were excluded from the experiment.

### Imaging Study Design

During each scan, fMRI data were acquired during five resting and five 2-back sessions, each lasting 4 minutes and 20 seconds. The resting sessions and working memory sessions were alternated. The participants were provided with fMRI compatible goggles, ear plugs, and a hand-fitted remote for the entirety of the procedure. During the rest condition, a black fixation cross was presented on the screen in the goggles for the entire duration of the scan. In the task condition, the participants were presented with a sequence of letters, each two seconds apart**.** The order of letters presented to each participant was randomly assigned in order to minimize the effect of the presentation sequence. The participants had to respond with an answer of yes or no on the hand-fitted remote as to whether the word presented on the screen was the same as the letter two before it. Each condition was presented a total of five times for each participant. Responses were recorded using E-prime 2.0 software. All anatomical and functional image processing was done using FSL, fMRIB software library [22]. Further processing for network statistics was completed using scripts performed on MATLAB.

### MR Image Acquisition

All imaging was performed on a Siemens SKYRA 3T MRI scanner. A foam padded birdcage head coil was utilized to limit artifacts that could occur as a result of head movement. The protocol parameters were the following: Whole-brain gradient echo echo-planar imaging (EPI) was used detect Blood-oxygen-level dependence (BOLD) fMRI signal changes during rest and the working memory task. The echo planar imaging contained the following parameters: 120 volumes with 35 contiguous slices per volume; slice thickness 5.0 mm; in-plane resolution of 3.5 mm×3.5 mm; TR/TE = 2000/25 ms. The voxels of each anatomical brain image were identified by using the AAL (Automatic Anatomical Labeling) atlas.

### Functional Image Pre-processing

The functional images were corrected for head motion by realigning them to the first image volume by using “rigid body” transforms. Next, the EPI image for each participant was co-registered, normalized to the standard stereotactic MNI (Montreal Neurological Institute) space, and resliced to 4.0 mm×4.0 mm×5.0 mm. The images were not smoothed in order to prevent creating local false correlations [15].

The time courses were extracted for each voxel in gray matter based on the Automated Anatomical Labeling atlas [23] and band-pass filtered to remove signals outside the range of 0.009–0.08 Hz [24], [25]. Mean global, white matter, and CSF signal as well as motion correction parameters were regressed from the filtered time series to account for physiological noise. A correlation matrix was then produced by computing correlations between all possible pairs of voxels. The correlation matrices were thresholded using the following N = K^S^, where S is the equivalent of the shortest path length in a random network, N = number of nodes, and K = degree. The correlation coefficient that satisfied N = K^S^ was used as a lower bound when creating binary adjacency matrices [25] This thresholding procedure ensures that the connection densities are consistent across conditions and individual subjects in the event that there is a change in the number of network nodes. Network properties were measured at various S values (2.5; 3.0; 3.5) and found to be independent of threshold effects (data not shown). The results presented here used S = 3.0 as a threshold.

### Network Metrics

Refer to Rubinov and Sporns [26] for a more detailed description of the metrics used in this study. Since local efficiency and global efficiency are comparable metrics and highly correlated with clustering and path length, we only present the results from local and global efficiency.

Degree, *K*, was calculated to determine the number of connections a node has to other nodes in the network. For each participant, a degree distribution was developed for each rest and 2-back run in order to show the probability of a degree in the entire network.

Global Efficiency, *E*_glob_, is the inverse of the path length, thus it measures how efficiently information spreads across the whole network. *E*_glob_ is a scaled measure that ranges from 0–1, with a value of 1 signifying maximum distributed processing.

Local Efficiency, *E*_loc_, is a measure of how efficiently information propagates through a node’s direct neighbors [9]. It is also a scaled measure ranging from 0–1, however a value of 1 signifies a node with solely local connections.

### Identifying and Quantifying Regions of Interest (ROIs) within Working Memory and Resting State Networks

The precuneus and dorsolateral prefrontal cortex, respectively, were found according to coordinates mentioned in previous literature [18]. Masks of these two regions of interest were created using WFU-pickatlas software [27]. The ROIs were generated using a 10 mm sphere placed at −9, −72, 37 for the left precuneus, 10, −69, 39 for the right precuneus, and +/−43, 22, and 34 for the dorsolateral prefrontal cortex. The ROIs were used to extract the average values of the specified network metrics from the whole brain network. First, the network metrics values were mapped to each voxel in brain space bases on the whole brain network analysis. This resulted in maps for each of the metrics of interest including global efficiency, local efficiency, and degree for each participant. The value of the network metric of interest was extracted from each voxel in the ROI. The values were then averaged to generate a mean for the ROI. The dorsolateral prefrontal cortex ROI contained 237 voxels and the precuneus ROI contained 233 voxels. Two (task) x five (session) mixed ANOVAs were then used to analyze the statistical significance of the effect of condition on each of these metrics within both the DLPFC and precuneus ROIs.
